# A Comparison of the Efficacy and Adverse Effects of Ketamine and Electroconvulsive Therapy in the Management of Treatment-Resistant Depression: A Systematic Review

**DOI:** 10.7759/cureus.55596

**Published:** 2024-03-05

**Authors:** Shaan I Chaudhri, Amina Amin, Binay K Panjiyar, Dhuha S Al-taie, Esraa M AlEdani, Jahnavi Gurramkonda, Pousette Hamid

**Affiliations:** 1 Psychiatry, California Institute of Behavioral Neurosciences & Psychology, Fairfield, USA; 2 General Surgery, Shifa International Hospital, Islamabad, PAK; 3 Global Clinical Scholars Research Training, Harvard Medical School, Boston, USA; 4 Internal Medicine, California Institute of Behavioral Neurosciences & Psychology, Fairfield, USA; 5 Plastic Surgery, California Institute of Behavioral Neurosciences & Psychology, Fairfield, USA; 6 Dermatology, California Institute of Behavioral Neurosciences & Psychology, Fairfield, USA; 7 Neurology, California Institute of Behavioral Neurosciences & Psychology, Fairfield, USA

**Keywords:** treatment of trd, trd, treatment-resistant depression, ect, ketamine

## Abstract

Ketamine has been repeatedly demonstrated to be an effective treatment in the management of patients with treatment-resistant depression (TRD). An important question is whether it is equally or more effective than the current gold standard of electroconvulsive therapy (ECT), as the adverse effects of ECT can lead to memory loss and neurocognitive deficits. A literature search was conducted for trials that directly compared the efficacy and adverse effects of ketamine and ECT via PubMed and Google Scholar. A total of 56 articles were identified with six included in this review. The studies included differed significantly in their quality and with differing levels of potential for bias. Ketamine has a more immediate effect when compared to ECT, but the antidepressant effects are shorter-lasting. Cognitive deficits were less pronounced in patients undergoing ketamine therapy. Many studies had a small number of participants and varied widely in the type of ECT used. Allocation bias seems likely in nonrandomized studies. Follow-up times were also short in some studies. The existing literature does not provide sufficient evidence to support the usage of ketamine over that of ECT for TRD, as remission rates were significantly higher over extended periods in ECT groups. Cognitive adverse effects were more pronounced in patients undergoing ECT. More high-quality randomized controlled trials (RCTs) directly comparing these two treatment modalities are required before drawing any firm conclusions.

## Introduction and background

Major depressive disorder (MDD) is one of the top ten largest causes of disease leading to disability-adjusted life years according to the World Health Organization. Despite MDD's ubiquitous nature and advances in drug therapies, it remains a significant challenge in the field of psychiatry. The root of this challenge is that up to 30% of patients diagnosed with MDD fail to show a response to trials of multiple antidepressants, resulting in a diagnosis of treatment-resistant depression (TRD) [1]. The current gold standard treatment for dealing with the challenges presented by TRD is electroconvulsive therapy (ECT) [2]. Despite its repeatedly proven efficacy, ECT faces some significant hurdles in its utilization due to misconceptions based on previous standards of treatment, contributing to a general reluctance to undergo so-called "shock therapy," a reductive colloquialism based on the public perception of the benefits of ECT. Many individuals believe that repeated use of anesthesia in ECT can cause retrograde amnesia, potentially extending back years, further contributing to hesitation in undergoing the procedure [3]. If a patient is willing to undergo ECT despite the potential side effects, variations in regional access may limit the ability of patients suffering from TRD to access adequate care as many regions may only have one psychiatrist qualified to perform ECT per million patients [4]. Additionally, most patients with TRD often relapse and require repeated sessions of ECT, potentially compounding the negative side effects [5]. Due to these barriers, there is a clear need for novel treatments for TRD that can achieve equal or higher rates of remission, with fewer hurdles to accessibility and a more favorable side effect profile.

One of the more promising avenues for potentially novel therapies for TRD is the use of N-methyl-D-aspartate (NMDA) receptor antagonists, particularly ketamine and its derivative, esketamine. Intranasally administered esketamine received approval from the FDA in 2019 for the treatment of TRD in adults when taken in conjunction with an antidepressant [6]. Because the dose of ketamine required to induce an antidepressant effect is smaller than the dose required for anesthesia, the side effect profile is generally mild following administration. Due to ketamine’s novelty in contrast to the repeatedly demonstrated efficacy of ECT, limited understanding of possible long-term side effects, and historical reputation as a street drug, some providers express reluctance in recommending ketamine to their patients suffering from TRD [7].

Given the need for readily available options for the treatment of TRD, the potential of ketamine compared to the established standard of ECT is worth examining. The possibility of any agent as, or even more, efficacious as ECT in treating TRD with less pronounced adverse effects and increased accessibility to treatment could lead to exciting new frontiers in the management of patients with debilitating TRD. The objective of this review is to synthesize the most recent available literature regarding the efficacy of ketamine as a therapeutic agent in the treatment of TRD compared to the current gold standard of ECT and provide a qualitative analysis to assist providers in making evidence-based decisions in the management of patients with this debilitating disease. Although ketamine is often used as an inductive agent for anesthesia, this review will focus on the use of ketamine or ECT for the treatment of TRD. Studies where ketamine was used in conjunction with ECT will not be included.

The comparative analysis of various studies assessing ketamine and ECT for treating TRD offers intriguing insights.

## Review

Methods

This systematic review was conducted by utilizing the Preferred Reporting Items for Systematic Reviews and Meta-Analyses (PRISMA) 2020 Guidelines [8]. An electronic search was conducted using PubMed and Google Scholar in May of 2023 utilizing the following search string ("Electroconvulsive Therapy" OR ECT OR "Electroshock Therapy") AND (Ketamine) AND (Depression OR "Major Depressive Disorder" OR "Clinical Depression") AND (Compare OR Comparison OR Outcome OR Comparative) as the search terms. After applying filters based on our inclusion and exclusion criteria, the articles were then reviewed by the authors, identifying studies that could potentially be used for conducting this review. Other possibly relevant studies in the references of these articles were also identified and included for further review by our authors.

Following the initial selection of articles based on our search criteria, we employed the Cochrane Risk-of-Bias (RoB) tool for randomized trials and the Risk Of Bias In Nonrandomized Studies-of Interventions (ROBINS-I) tool for nonrandomized studies. This rigorous assessment allowed us to evaluate the potential biases in each study, including selection, performance, detection, attrition, and reporting biases in the case of randomized trials, and confounding factors, selection of participants, classification of interventions, deviations from intended interventions, missing data, measurement of outcomes, and selection of reported result in nonrandomized studies. The application of these tools ensured that only studies with a low to moderate RoB were included in our review. This step was critical in maintaining the integrity and scientific rigor of our systematic review, as it allowed us to include only those studies that met our stringent criteria for methodological soundness and reliability.

Criteria of Eligibility

The eligibility of studies for this systematic review was determined following the Population, Intervention, Comparison, Outcome, and Study (PICOS) Framework [9]. (1) Population: adult patients with unipolar or bipolar treatment-resistant depression. (2) Intervention: administration of ketamine. (3) Comparison: administration of any form of ECT. (4) Outcome: remission of treatment-resistant depression, adverse effects. (5) Studies: randomized controlled trials (RCTs) or cohort studies published in English. Case reports, case series, reviews, case-control studies, and letters were omitted from our results as were studies not published in the English language. Any articles published before 2012 were excluded to ensure that any information would be as current and relevant as possible. Articles that used ketamine as an adjunctive agent to ECT were also excluded from this review.

Results

Our initial search yielded 8944 results, after using Google’s advanced search; these results were filtered into 56 potentially relevant articles. These articles were then manually reviewed for their relevance. Many of our results dealt with ketamine as an adjunctive agent as an anesthetic agent, an interesting topic worthy of further consideration but irrelevant to this search.

**Figure 1:**
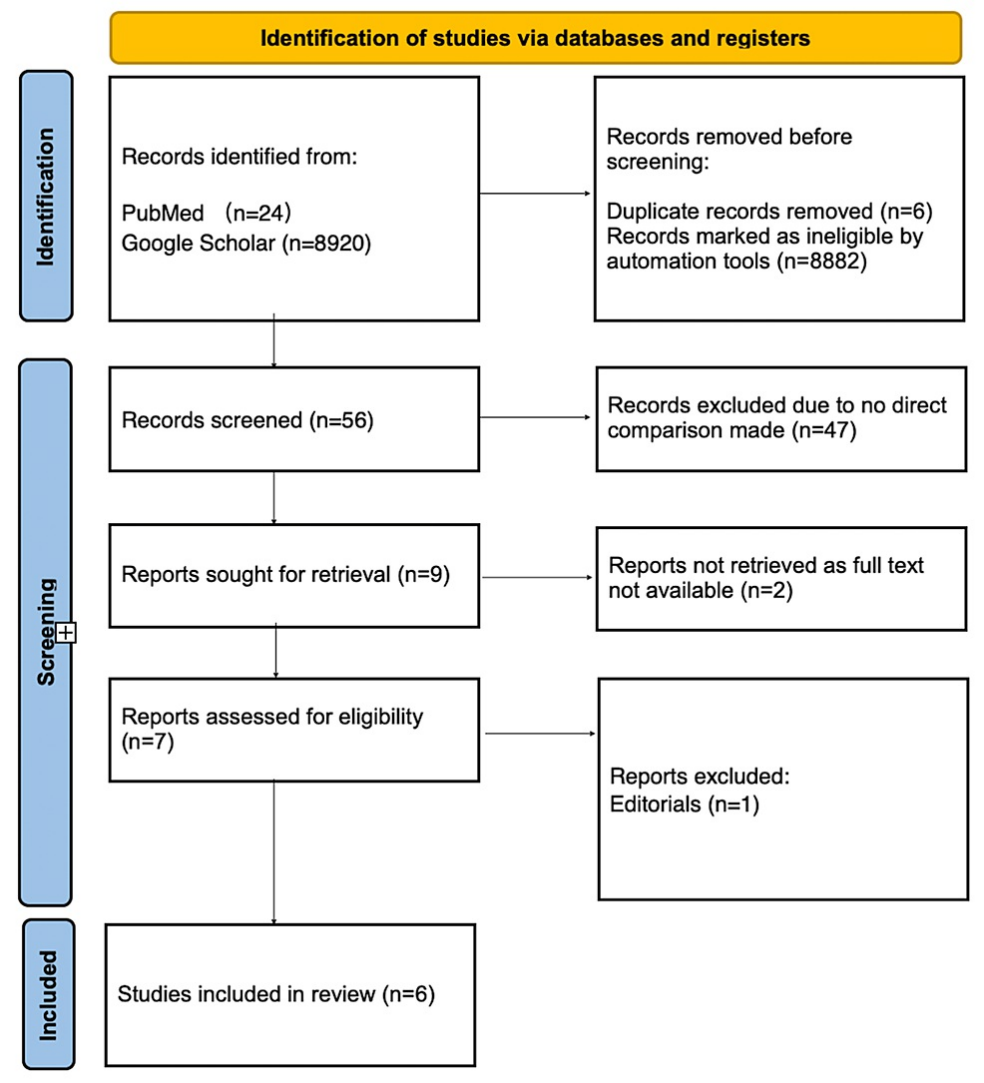
The PRISMA diagram PRISMA: Preferred Reporting Items for Systematic Reviews and Meta-Analyses

After assessing articles for their eligibility using our inclusion and exclusion criteria, we were left with six RCTs; of which two were naturalistic trials. All trials included directly compared ECT and ketamine in the treatment of TRD, compared the effect of the intervention on depression, and examined the most serious adverse effects experienced by each group based on the intervention they received. A flowchart of the process of selection according to PRISMA guidelines, including reasons for the exclusion of any articles, is included in Figure 1.

**Figure 2:**
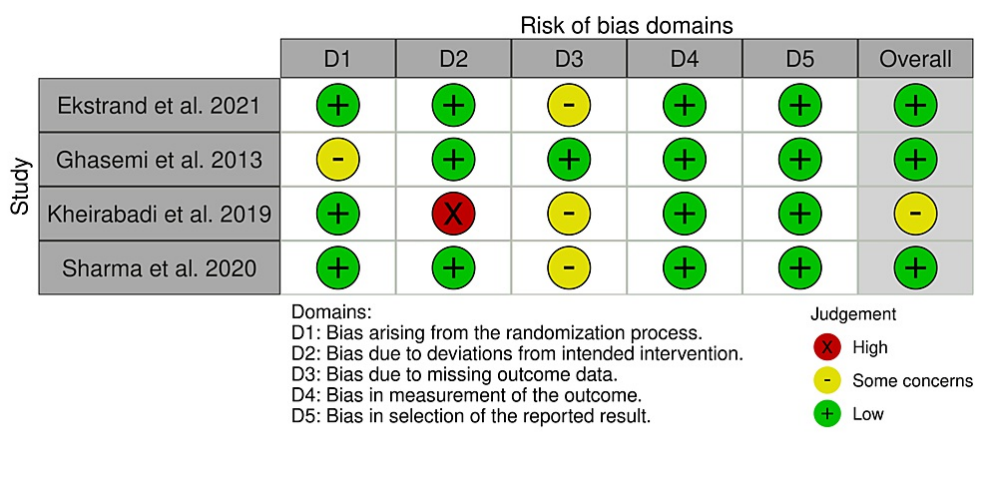
RoB2 risk of bias assessment RoB2:  Version 2 of the Cochrane risk-of-bias tool for randomized trials

**Figure 3:**
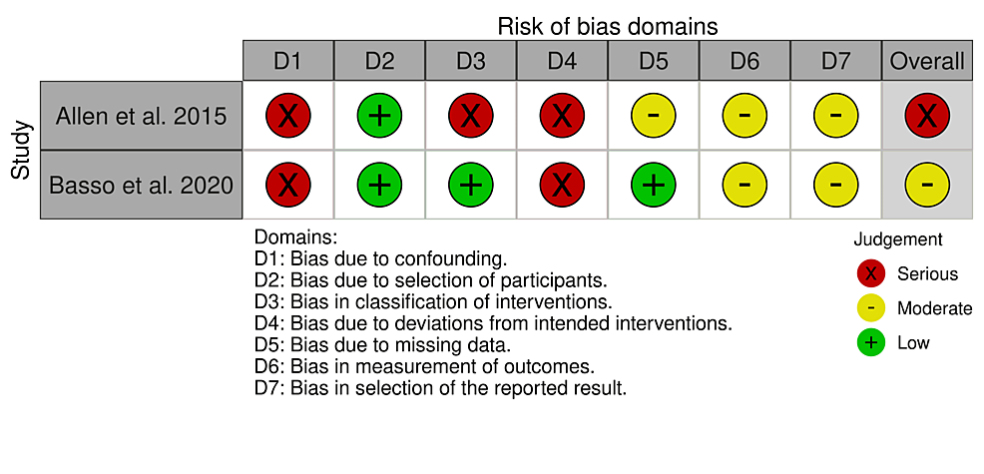
ROBINS-I risk of bias assessment ROBINS-I: Risk Of Bias In Nonrandomized Studies-of Interventions

We then used the Cochrane RoB tool to determine the presence of any potential bias in these RCTs that may have had a tangible effect on the findings included in this study. This analysis looked at seven domains of each trial to ascertain whether the RoB based on the domain could be classified as high, medium, or low RoB [10]. For non-RCT studies, we used the RoB in ROBINS-I tool [11]. Figure 2 and Figure 3 below contain the results of these checks.

After examining for any potential bias, data extraction was performed by our researchers. Study design, population, intervention, effect of intervention on TRD, and adverse effects were all recorded and analyzed for further discussion. The results of this analysis are recorded in Table 1.

**Table 1:** Relevant information from collected studies MADRS: Montgomery-Åsberg Depression Rating Scale; HDRS: Hamilton Depression Rating Scale; RCT: randomize controlled trial; ECT: electroconvulsive therapy; DSST: digit symbol substitution test; B4ECT-ReCoDe; battery for ECT-related cognitive deficits; N: number of participants

Study	Design	Number of participants	Antidepressant effects	Reported adverse effects
Ghasemi et al. [12]	Blinded RCT	18	HDRS scores were reduced significantly in the ketamine group after two treatments but were not significantly different after the third treatment.	According to the authors, the effects of ECT and ketamine were both well-tolerated.
Kheirabadi et al. [13]	Blinded RCT	32	HDRS scores were not significantly different in both groups until after the final treatment, where ECT patients had significantly lower scores following one week after the sixth treatment (p = 0.5), one month after (p = 0.3), two months after (p = 0.1), and three months after (p = 0.4).	Cognition was less affected by ketamine therapy. Ketamine adverse effects-dizziness (100%), headache (60%), blurry vision (60%), numbness of half body (60%), depersonalization (60%), vertigo (40%), diplopia (40%), nausea (30%), nystagmus (5%), and increased respiration and heart rate (5%); ECT adverse effects-headache (100%), dizziness (92%), muscle pain (92%), nausea (75%), joint pain (50%), orientation disorder (33%), and extended seizure (5%).
Ekstrand etal. [14]	Randomized Open-Label Trial	186	The authors concluded that ECT was superior to ketamine and produced higher rates of remission (63% ECT vs. 46% ketamine) (p = 0.026). Final MADRS scores were significantly lower in the ECT group (ECT: 12.2 ± 11.1; ketamine: 16.9 ± 13.1; P = .009).	A total of 21 patients from the ketamine group and four patients from the ECT group were forced to withdraw. ECT patients reported significantly more headaches, muscle pain, and amnesia and twice as often suffered side effects lasting 24 hours or longer (ECT: 48/90; ketamine: 20/91, chi2 = 18.5).
Sharma et al. [15]	Blinded RCT	26	Significant improvement on HDRS scores in both groups after treatment when compared to baseline (p < 0.001).	Significant improvement on the DSST variable of the B4ECT-ReCoDe compared to baseline (p = 0.017) in the ketamine group.
Basso et al. [16]	Open Label	46	MADRS scores were reduced more from T0 to T1 with no significant difference until after T2.	Cognitive differences were found with attention; verbal memory and executive function decreased in ECT patients (p≤.05).
Allen et al. [17]	Open Label	34	Ketamine (p < 0.001) and ECT (p = 0.001) significantly reduced HDRS scores compared to baseline.	Ketamine elevation of diastolic BP (n = 1); unpleasant experience (n = 2).

Discussion

The first study we examined, Ghasemi et al. [12], involved a single-blinded randomized trial with 18 patients divided into two groups: one receiving ketamine (n = 9) and the other undergoing ECT (n = 9) therapy for TRD. Patients in the ECT group underwent three sessions at 48-hour intervals, totaling three sessions, while those in the ketamine group received 0.5 mg/kg mL of ketamine in three sessions spaced 48 hours apart. The Hamilton Depression Rating Scale (HDRS) assessed treatment efficacy after each session. Following the final treatment, ECT patients exhibited a mean HDRS score of 14, notably reduced from the baseline of 35.8 after one week. Similarly, ketamine-treated patients showed a significant symptom decrease, with HDRS scores declining from 30.22 to 9.55 a week post-final treatment. Ketamine notably outperformed ECT in reducing the HDRS scores after the initial two treatments. After the first treatment, ketamine patients averaged a score of 16.88 on the HDRS, significantly lower than the mean baseline HDRS of 34.66 (p = 0.00). Conversely, following the initial ECT session, patients scored 31.44, displaying a comparatively modest reduction from the mean baseline of 35.88, which was statistically significant (p = 0.000). Both ECT and ketamine were well-tolerated by patients, showing no significant differences in hemodynamic parameters posttreatment. Consequently, the authors concluded that ketamine exhibits greater effectiveness than ECT, especially in patients with MDD lacking psychotic features, and elicits a quicker response to treatment [12]. A major limitation of this study is the lack of long-term follow-up, as previously mentioned. Thus, future practice should consider ketamine's rapid response and effectiveness in treating MDD without psychotic features compared to ECT, as demonstrated in this study. Emphasizing ketamine's potential after initial treatments and its tolerability suggests its utility as a viable alternative or adjunct therapy for TRD management.

The second study by Kheirabadi et al. [13] was a double-blinded RCT involving 32 patients split equally between ketamine and ECT groups. ECT involved bitemporal stimulation, while ketamine was infused at 0.5 mg/kg with saline over 40 minutes. The HDRS scores were tracked at baseline, before each of six sessions, and during follow-ups up to three months. Ketamine showed faster HDRS reduction, though scores returned to baseline after a month, while ECT displayed lower HDRS scores for three months posttreatment. Differences weren't significant (p = 0.05) likely due to patient numbers. ECT patients reported more headaches, joint, and muscle pain; ketamine patients experienced blurry vision, vertigo, diplopia, numbness, and depersonalization. WMS scores concerning memory in ECT patients were lower after one week and one month, but not significantly. The study suggests that ketamine matches ECT's effectiveness in depression treatment and might offer advantages in reducing posttreatment memory deficits. Limitations included a small patient sample and unclear treatment blinding [13].

In contrast, the third RCT, Ekstrand et al. [14], a randomized open-label non-inferiority trial, stands out as the most comprehensive among the studies in this review. The primary outcome measured was remission of depression, as defined by a score of 10 on the Montgomery-Asberg Depression Rating Scale (MADRS). The secondary outcomes included adverse events, time to remission, and time to relapse. All 186 participants who received at least one of the two therapy options were included in the aggregated results. All participants were followed up with for 12 months following the final treatment session. Participants in the ECT control group received up to 12 sessions of therapy or were treated until remission was achieved. In the ECT group (n = 91), 57 out of 91 (63%) patients remitted entirely following completion of their therapy, as defined above by scores of <10 on MADRS, compared to the ketamine group (n = 95) where only 44 of the patients (46%) were found to experience remission from their depressive symptoms. This, combined with lower MADRS scores at the end of patient treatments, led the researchers to conclude that ketamine therapy could not be considered non-inferior to ECT. Furthermore, relapse rates after 12 months in the ECT group (64%) were also lower than in the ketamine group (70%), though no significant differences were found in the amount of time to relapse. Adverse effects were extensively reported with ECT responsible for significantly more amnesia, headaches, and muscle pains (P < 0.001). ECT was responsible for prolonged amnesia in 21 patients, with three patients experiencing amnesia 12 months after treatment. Ketamine was significantly responsible for euphoria, dissociation, vertigo, diplopia, blurred vision, and anxiety (P = 0.001 for all). The authors of this study conclude that ketamine is non-inferior to the treatment of TRD in comparison to ECT; however, ECT produces enough of a difference in remission to still be considered the gold standard. ECT was also found to be significantly more effective in elderly patients [14]. This study was unique among those compiled here due to its relatively longer follow-up period.

In the fourth and final blinded randomized trial by Sharma et al. [15], 0.5 mg/kg mL ketamine infusions (n = 13) was compared against a right-unilateral or bifrontal ECT (n = 13) in 26 patients over six alternate-day sessions. The HDRS scores in both groups were comparable at baseline before treatment was initiated. In this study, patients undergoing ECT experienced greater reductions in the HDSR scores, quicker response rates to treatment, and sustained remission of symptoms in up to 92.3%. In contrast to ECT, patients of the ketamine group only experienced remission in 50% of reported cases. This was deemed by researchers as being significant (P = 0.030). Cognitive effects were measured by the Battery for ECT-Related Cognitive Deficits (BERCD) administered to both groups, and patients in the ketamine group scored higher indicating less severe memory deficits compared to ECT where BERCD scores were not significantly different before or after undergoing the procedure. This effect, however, was only found to be significant in one variable that composes the BERCD and the DSST, where scores in the ketamine group were found to be significantly higher (P = 0.017). The authors credit this disparity to the inability of the patients being treated to properly respond to the cognitive tests administered because of their illness. The reported remission rates for ECT in this trial are uncommonly high compared to the existing literature, possibly due to the number of patients involved [15]. An important limitation of this study is that the cognitive effects of ECT were not stratified based on the method of ECT treatment that they received.

Due to the limited number of studies meeting our search criteria, we opted to include high-quality naturalistic studies that aligned with our criteria in this analysis. The first such naturalistic review by Basso et al. [16] was a nonrandomized trial, where participants received either right unilateral ECT treatments over the course of four weeks (n = 22), 0.5 mg/kg IV infusions of ketamine over a two-week period (n = 24), or even both depending on response to treatment. This “real-world” assessment of the effectiveness of these two therapies used the MADRS to quantify the reduction of depressive symptoms, and a score of <10 was characterized as remission. Ketamine produced a significantly faster response in treated patients than ECT, and midtreatment MADRS scores were lower for the ketamine group as well. At the end of four weeks, however, the difference in response to treatment in both groups was not statistically significant. A battery of neurocognitive tests was also administered to patients in both treatment groups. The effects of ECT and ketamine were significantly different from one another in the domains of verbal memory, attention, and executive function with patients undergoing ketamine therapy scoring higher in those domains. Deficits in immediate memory and visual memory were not significant. There were several large limitations of this study, there was no control group, and patients were allowed to choose between ECT and ketamine. As a result of the stigma against ECT, the authors acknowledge that this choice presented a potentially confounding event and may have contributed to the rapid response to ketamine [16].

Our second study of this nature by Allen et al. [17] was an experimental study measuring the potential effects of ECT or ketamine therapy on a novel biomarker, serum brain-derived neurotrophic factor (sBDNF), which is currently theorized to be a biomarker that may aid in identifying and treating depression. Levels are lower in patients with treatment-resistant depression and have been observed to rise in patients who have achieved remission of their depressive symptoms. Seventeen patients were given three infusions of 0.5 mg/kg ketamine, while 17 more were treated with twice weekly brief-pulse bitemporal ECT. Patients undergoing ECT showed a delayed but sustained decrease in their HDRS scores, as ketamine responders in this study once again showed a rapid response, based on HDRS, but quickly returned to baseline levels of depression. The responders to ketamine therapy did have normalization of their sBDNF serum levels compared with normal controls, in contrast to ECT which showed no difference in sBDNF levels post-ECT in patients with remission of symptoms. The differences between ECT and ketamine therapy on the HDRS posttreatment were ultimately not found to be statistically significant by the authors. Both treatments were, however, found to significantly reduce HDRS scores. There were seven total dropouts from the ketamine group, with only three directly related to therapy. Two of those patients reported unpleasant side effects, and one was forced to withdraw due to an unsafe rise in diastolic blood pressure. No patients dropped out from the ECT comparison group. Once again, the follow-up duration was not long enough, leading to issues when trying to predict whether this disparity in sBDNF would still be present following a more thorough course of ECT treatment or prolonged ketamine infusion protocols [17].

Although the available data is limited and not enough to support any strong conclusions, some inferences can be made from the reviewed articles. It is also pertinent that the results of all studies were measured using subjective depression rating scales. This presents some concerns of bias, but results were consistent across studies. Both ketamine and ECT demonstrate consistent efficacy across all studies when used for the treatment of TRD. For patients with TRD who are reluctant to undergo ECT, ketamine may present an alternative that offers relief. The response to ketamine appears to be more immediate and may offer utility in the treatment of acute depressive episodes where an urgent response to treatment is desired. ECT appears to sustain remission for longer periods than ketamine, potentially leading to more required treatments for amelioration of depression, as seen in Kheirabadi et al., Ghasemi et al., and Ekstrand et al. [12-14]. This could be an important limitation to consider when dealing with patients with limited access to potential therapeutic options. Both ketamine and ECT used in combination may be an avenue worthy of future consideration and research.

Adverse effects were common in both treatment groups across studies. Memory loss, a commonly reported side effect of ECT, was reported across all studies. The severity of deficits differed between studies, but amnesia was reported in Ekstrand et al., Sharma et al., and Kheirabadi et al. [13-15]. Dissociation and derealization were commonly reported in candidates undergoing ketamine therapy. This may cause discomfort for patients who experience those sensations because of their depression. These patients may be particularly ill-suited for ketamine therapy, although further study is required for more firm conclusions. The commonly reported side effect of ketamine-induced hypertension was not common in the studies that included the doses required to produce a pharmacological response. Overall, despite the common perceptions of ECT, both treatments were adequately tolerated across groups. Ketamine should be considered an effective option in patients who find their memory loss distressing.

To conclude, the studies by Ghasemi et al. [12], Kheirabadi et al. [13], Ekstrand et al. [14], Sharma et al. [15], Basso et al. [16], and Allen et al. [17] offer diverse insights into ketamine and ECT for TRD. Ghasemi et al. highlight ketamine's rapid response compared to ECT after initial treatments. Kheirabadi et al. indicate similar efficacy between ketamine and ECT but with different side effects and memory outcomes. Ekstrand et al. present ECT as superior for remission and elderly patients, contrasting with ketamine's lower remission rates. Sharma et al. show ECT's greater efficacy for remission but acknowledge ketamine's potential cognitive advantages. Basso et al. and Allen et al. provide "real-world" assessments and explore novel biomarkers, showing varied responses between ECT and ketamine with limitations in study design and follow-up duration.

Limitations

One of the major limitations of this study was the small number of high-quality RCTs that have so far been performed directly comparing ketamine and ECT; often the ones that do exist have differing conclusions. More RCTs that make a direct comparison between the outcomes of ketamine and ECT are needed to ascertain the efficacy of ketamine in patients with TRD. Since some outcomes were reported via standardized rating scales for depression, the potential for bias among patients self-reporting the severity of their symptoms exists. Another important limitation in the applicability of these results is whether patients were continued or initiated on pharmacological therapy as well. It is unclear, due to the limited nature of the current literature, whether ketamine enhances the antidepressant effects of pharmacological agents or if ketamine itself is responsible for the reduction in self-reported depressive symptoms. A common limitation stated by the authors above is the inability to effectively blind patients to the treatment option being offered to them, as the two differ significantly in their methods.

While ketamine shows promise in the treatment of TRD and could be considered a therapeutic agent in patients who fail to respond to ECT, these findings are limited by the lack of reliable controlled trials directly comparing the two treatment modalities. In general, cognitive adverse effects were significantly lower in patients who received ketamine which may make ketamine a viable option in patients with significant distress as a result of memory loss from ECT. If further studies confirm the efficacy of ketamine in treating TRD at levels comparable to ECT, with less severe cognitive deficits after therapy, NMDA-receptor antagonists could potentially lead to new avenues for treating this crippling illness with less adverse outcomes for patients involved in the treatment, especially in patients with acute suicidality where a rapid response to treatment is desired. Ketamine also shows some promise as an adjunctive agent in traditional ECT, typically in its traditional form as an anesthetic agent. This could also prove useful in treating acute suicidality in locales where ECT is not easily available, as ketamine is a commonly used anesthetic agent in wide circulation. Any benefit derived from ketamine should also be measured against the yet still unknown side effects of repeated dosages of ketamine therapy over a sustained period.

## Conclusions

Ketamine has repeatedly been shown to be effective in the treatment of treatment-resistant depression; however, efficacy in comparison to ECT is still ambiguous. In situations where ECT is unavailable or the adverse side effects make continuing treatment unviable, ketamine may serve as a rapid and effective mechanism for treating TRD and acute suicidality. However, the effects of ketamine appear to be less sustained over longer periods of time following the last treatment, and some patients may fail to show a response. Further research is needed before drawing any firm conclusions, especially with regard to any possible long-term side effects in the usage of ketamine. Despite the misperceptions of ECT, the side effects of amnesia and muscle soreness were often present in the abovementioned studies but were not severe enough to prevent most patients experiencing these symptoms from seeking further treatment to experience relief of their depressive symptoms. This is fairly in line with the current understanding of the risks and benefits of ECT. Ketamine therapy, while not responsible for amnesia or muscle soreness, does cause some patients distress due to the unpleasant adverse effect of dissociation. This dissociative effect is an inherent property of ketamine and may limit its effectiveness in treating certain patients with TRD. No long-term studies examining the side effects of ketamine as a treatment in TRD currently exist, in stark contrast to the plethora of literature over decades that has explored the consequences of sustained administration of shock therapy and reinforced its status as the gold standard for treatment of TRD. Ketamine, however, has a far more favorable neurocognitive adverse effect profile in comparison to ECT.
